# Supplementary material for: Evaluation of New Potential Inflammatory Markers in Patients with Nonvalvular Atrial Fibrillation
Source: Int J Mol Sci. 2023 Feb 7;24(4):3326. doi: 10.3390/ijms24043326 (PMC9964249; doi:10.3390/ijms24043326)
Supplement: Supplementary file 1 [file ijms-24-03326-s001.zip › ijms-2080725-supplementary.pdf]

Supplementary Material

Supplementary Table S1. Plasma levels of inflammatory mediators comparing controls and NVAF patients.

**Inflammatory mediators (pg/mL)**	**Controls (N = 50)**	**NVAF (N = 55)**	***p* value**
IL-2	1.26 (0.37)	3.04 (1.38)	**< 0.001**
IL-4	1.21 (0.30)	2.99 (1.63)	**< 0.001**
IL-6	1.72 (1.05)	7.96 (8.78)	**< 0.001**
IL-10	0.87 (0.44)	2.40 (1.30)	**< 0.001**
TNF	1.25 (0.44)	3.06 (1.60)	**< 0.001**
IFN-γ	1.12 (0.39)	2.86 (1.44)	**< 0.001**
IL-8	3.60 (1.60)	3.43 (2.14)	0.182
RANTES	2031.36 (2106.59)	2331.56 (2088)	0.987
MIG	94.45 (91.39)	160.95 (184.65)	**0.032**
MCP-1	59.73 (42.50)	65.60 (65.65)	0.902
IP-10	105.72 (74.20)	128.67 (110.38)	**0.008**
TGF-β	1.68 (0.86)	1.48 (0.75)	0.476
ADAMTS13	2.01 (0.64)	2.18 (1.13)	0.226
GDF-15	0.01 (0.02)	0.03 (0.09)	**0.006**
Myoglobin	0.58 (0.66)	0.61 (0.49)	0.953
sICAM-1	115.74 (1166.96)	414.88 (1515.95)	0.070
MPO	0.20 (0.34)	0.25 (0.42)	**0.005**
p-selectin	0.25 (0.18)	0.28 (0.07)	0.340
NGAL	1.28 (0.84)	1.49 (0.83)	**0.042**
sVCAM-1	6.22 (4.68)	6.86 (4.84)	0.594
SAA	82.85 (214.81)	176.78 (1256.82)	**0.027**

Mann-Whitney *U* test were performed for non-normal continuous variables (values expressed as median and interquartile range). ADAMTS13 = disintegrin and metalloproteinase with thrombospondin type 1 motif, 13; GDF = growth differentiation factor; IFN-γ = Interferon-gamma; IL = interleukin; IP-10 = interferon-gamma-induced protein; MCP = monocyte chemoattractant protein; MIG = monokine induced by interferon-gamma; MPO = myeloperoxidase; NGAL = neutrophil gelatinase-associated lipocalin; RANTES = regulated on activation, normal T cell expressed and secreted; SAA = serum amyloid A; sICAM = soluble intercellular adhesion molecule; sVCAM-1 = soluble vascular cell adhesion protein; TGF-β = transforming growth factor-beta; TNF = tumor necrosis factor. Statistically significant differences between groups are indicated in bold fonts, with a significance level of $p < 0.05$.

Supplementary Table S2. Univariate logistic regression for inflammatory mediators.

**Inflammatory mediators (pg/mL)**	**OR (95% CI)**	***p* value**
IL-2	3.63×10^5 (91.15-1.45 x 10^9)	**0.002**
IL-4	7.05×10^3 (80.53-6.19 x 10^5)	< **0.001**
IL-6	1.74 (1.36-2.22)	< **0.001**
IL-10	13.34 (4.85-36.7)	< **0.001**
TNF	11.03 (4.41-27.6)	< **0.001**
IFN-γ	8.74×10^3 (89.87-8.5 x 10^5)	< **0.001**
MIG	1.01 (0.99-1.0)	0.346
IP-10	1.01 (1.00-1.01)	**0.016**
GDF-15	49.59 (0.18-1.41 x 10^4)	0.176
MPO	0.97 (0.73-1.28)	0.834
NGAL	1.43 (0.77-2.65)	0.255
SAA	1.00 (1.00-1.00)	0.457

CI = confidence interval; GDF = growth differentiation factor; IFN-γ = Interferon-gamma; IL = interleukin; IP-10 = interferon-gamma-induced protein; MIG = monokine induced by interferon-gamma; MPO = myeloperoxidase; NGAL = neutrophil gelatinase-associated lipocalin; OR = odds ratio; SAA = serum amyloid A; TNF = tumor necrosis factor. Statistically significant differences are indicated in bold fonts, with a significance level of $p < 0.05$.

Table S3. Validity of multivariate logistic regression models.

	**Model 1**				**Model 2**			
**Inflammatory mediators (pg/mL)**	χ^2	**df**	***p* value**	**R^2 Nagelkerke**	χ^2	**df**	***p* value**	**R^2 Nagelkerke**
IL-2	134.975	5	< **0.001**	0.970	128.915	10	< **0.001**	1.000
IL-4	126.114	5	< **0.001**	0.938	128.915	10	< **0.001**	1.000
IL-6	67.690	5	< **0.001**	0.639	87.653	10	< **0.001**	0.814
IL-10	72.542	5	< **0.001**	0.670	68.010	10	< **0.001**	0.692
TNF	77.671	5	< **0.001**	0.702	79.315	10	< **0.001**	0.765
IFN-γ	130.408	5	< **0.001**	0.954	128.915	10	< **0.001**	1.000
IP-10	40.672	5	< **0.001**	0.432	46.941	10	< **0.001**	0.528

IFN-γ = Interferon-gamma; IL = interleukin; IP-10 = interferon-gamma-induced protein; TNF = tumor necrosis factor. Statistically significant differences are indicated in bold fonts, with a significance level of $p < 0.05$.
